# Automated Detection and Recognition of Wildlife Using Thermal Cameras

**DOI:** 10.3390/s140813778

**Published:** 2014-07-30

**Authors:** Peter Christiansen, Kim Arild Steen, Rasmus Nyholm Jørgensen, Henrik Karstoft

**Affiliations:** Department of Engineering, Aarhus University, Finlandsgade 22, Aarhus, Denmark; E-Mails: kim.steen@eng.au.dk (K.A.S.); rnj@eng.au.dk (R.N.J.); hka@eng.au.dk (H.K.)

**Keywords:** thermal imaging, feature extraction, kNN, DCT, pattern recognition

## Abstract

In agricultural mowing operations, thousands of animals are injured or killed each year, due to the increased working widths and speeds of agricultural machinery. Detection and recognition of wildlife within the agricultural fields is important to reduce wildlife mortality and, thereby, promote wildlife-friendly farming. The work presented in this paper contributes to the automated detection and classification of animals in thermal imaging. The methods and results are based on top-view images taken manually from a lift to motivate work towards unmanned aerial vehicle-based detection and recognition. Hot objects are detected based on a threshold dynamically adjusted to each frame. For the classification of animals, we propose a novel thermal feature extraction algorithm. For each detected object, a thermal signature is calculated using morphological operations. The thermal signature describes heat characteristics of objects and is partly invariant to translation, rotation, scale and posture. The discrete cosine transform (DCT) is used to parameterize the thermal signature and, thereby, calculate a feature vector, which is used for subsequent classification. Using a k-nearest-neighbor (kNN) classifier, animals are discriminated from non-animals with a balanced classification accuracy of 84.7% in an altitude range of 3–10 m and an accuracy of 75.2% for an altitude range of 10–20 m. To incorporate temporal information in the classification, a tracking algorithm is proposed. Using temporal information improves the balanced classification accuracy to 93.3% in an altitude range 3–10 of meters and 77.7% in an altitude range of 10–20 m

## 1. Introduction

In agricultural mowing operations, thousands of animals are injured or killed each year, due to the increased working widths and speeds of agricultural machinery. Several methods and approaches have been used to reduce this wildlife mortality. Delayed mowing date, altered mowing patterns (e.g., mowing from the center outwards [1]) or strategy (e.g., leaving edge strips), longer mowing intervals, the reduction of speed or higher cutting height [1] have been suggested to reduce wildlife mortality rates. Likewise, searches with trained dogs prior to mowing may enable the farmer to remove, e.g., leverets and fawns to safety, whereas areas with bird nests can be marked and avoided. Alternatively, various scaring devices, such as flushing bars [1] or plastic sacks set out on poles before mowing [2], have been reported to reduce wildlife mortality. However, wildlife-friendly farming often results in lower efficiency. Therefore, attempts have been made to develop automatic systems capable of detecting wild animals in the crop without unnecessary cessation of the farming operation. For example, a detection system based on infrared sensors has been reported to reduce wildlife mortality in Germany [3]. The disadvantage of the system proposed in [3] is its low efficiency, as the maximum search power is around 3 ha/h, when the weather conditions are fit.

In the [4], principles from [3] were further developed and tested. They conclude that vision systems are not a viable solution when the cameras are mounted on the agricultural machinery, as image quality is highly affected by the speed and vibrations of the machine. Instead a UAV-based system is utilized [5]. Using this solution, the movement of the tractor does not affect the image quality, and it is possible to manually scan large areas. The authors show that thermal imaging can be used to detect roe deer fawns based on aerial footage. However, the detection is performed manually and should be automated to increase efficiency. They conclude that the thermal imaging strategy is sensitive to the detection of false positives, meaning that objects that are heated by the Sun are falsely labeled (manually) as roe deer fawns.

UAVs are an emerging technology, and in modern agriculture, it can be utilized for many purposes. The UAV technology is capable of performing advanced and high precision tasks, due to the flight capabilities and the possibility to equip the aerial vehicle with computers and sensors, including thermal cameras. During the last two decades, thermal imaging has gained more and more attention in computer vision and digital image processing research and applications. Thermal imaging has become an interesting technology in outdoor surveillance, pedestrian detection and agriculture, due to the invariance to illumination and the lowered price of thermal cameras [6].

In [7,8], thermal imaging is used for person detection. The authors present thermal images of people at different times of the day and during summer and winter. Here, it is clear that the object of interest (people) does not always appear brighter (higher temperature) than the background. They propose background subtraction techniques, followed by a contour-based approach to detect people in the thermal images. Background subtraction is also utilized in [9–11]. However, this approach is not suitable for our UAV-based application with non-stationary cameras, as the background changes rapidly over time, and it is not possible to construct a background image. Another approach is the detection of hot spots based on a fixed temperature threshold [12–15]. In [16], a probabilistic approach for defining the threshold value is presented; however, it is still a fixed value.

There is little research within the automatic detection and recognition of animals in thermal images. Most research with thermal cameras involve static cameras, where background subtraction has been used for robust people detection in thermal images. In [5], a UAV, equipped with a thermal camera, is used for the detection of roe deer fawns in agricultural fields. Detection is based on manual visual inspection, and the author utilizes automatic gain control to enhance the appearance of living objects. An algorithm for the classification of roe deer fawns in thermal images is presented in [17]. They utilize normalized compression distance as the features followed by a clustering algorithm for classification. The dataset consists of 103 images, with 26 containing fawns hidden in grass. The same dataset is used in [18], where fast compression distance is applied in the feature extraction step and a nearest neighbor classifier is used for classification. In both papers, the features are derived from a dictionary, generated by a compression algorithm. These features are scale invariant; however, they are not rotation invariant, and they rely on absolute temperature measurements, which could be invalidated if animals are heated by the Sun. An algorithm for automatic detection of wildlife in agricultural fields is presented in [19]. However, the distinction between animals and other hot objects is not a part of the results presented. An algorithm for the identification of deer, to avoid deer-vehicle crashes, is presented in [20]. The histogram of oriented gradient (HOG) is used for feature extraction, and support vector machines are utilized in the classification step. Their method relies on occlusion-free side-view images and performs poorly if these criteria are not met.

This paper presents a method for detecting and recognizing animals in thermal images. The method is based on a threshold, dynamically fitted for each frame, and a novel feature extraction algorithm, which is invariant to rotation, scaling and, partly, posture. Detected objects are tracked in subsequent images to include temporal information within the recognition part of the algorithm. The algorithm has been tested in a controlled experiment, using real animals, in the context of wildlife-friendly farming.

## 2. Materials and Methods

**Figure 1.**
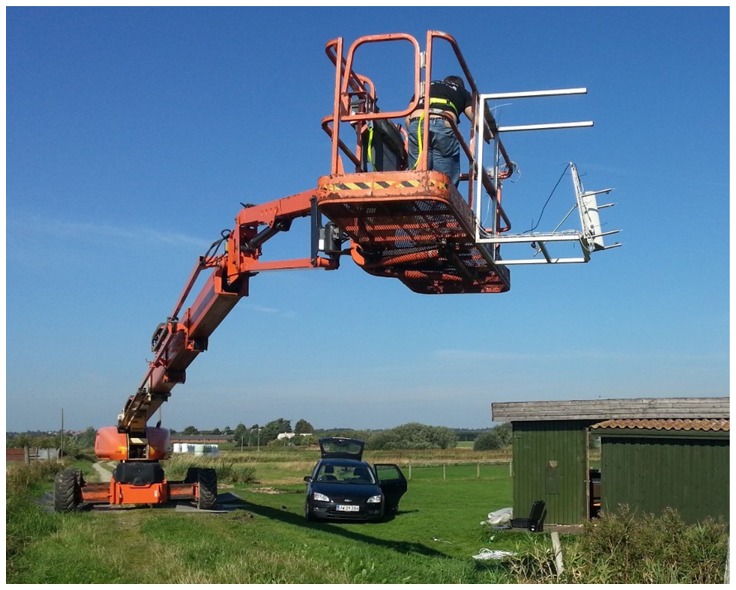
The setup used for capturing visual RGB and thermal images.

A telescopic boom is used to capture top-view images above a stationary scene, as shown in Figure 1. By using a telescopic boom lift, images can be captured at different altitudes, thus simulating the UAV. Unlike, using a UAV, the captured images are not affected by wind or vibrations within the UAV, which could affect image quality. Furthermore, the setup also avoids the compression of data, which might degrade data quality with respect to classification.

### 2.1. Data

A rig with a thermal and a regular RGB camera is mounted on the lift, recording 9 frames per second with a resolution of 320 × 240 and 1624 × 1234, respectively. Animals and four halogen spotlights (used as reference points) were manually placed below the lift within the field-of-view of the two imaging sensors. The altitude of the cameras was measured with a GPS. A total of six recordings were made through two days with temperatures of 15−19 °C and 16−23 ° C respectively. The recordings were captured using different areas around the scene shown in Figure 1.

**Figure 2.**
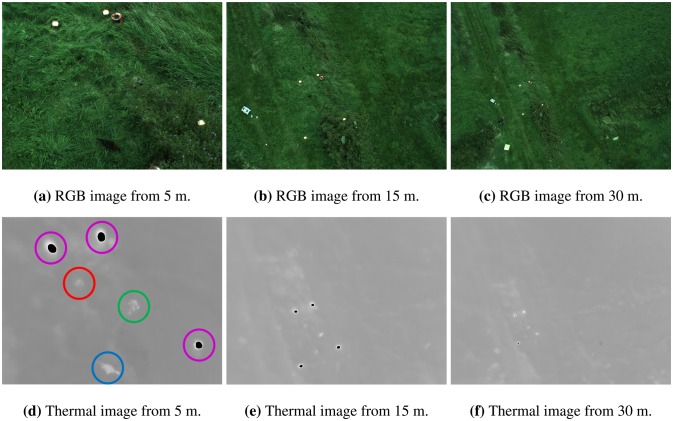
Visual RGB and thermal images capture the same scene from 5 m (**a**), 15 m (**b**) and 30 m (**c**). The scene consists of four halogen spotlights, a molehill, a rabbit and a chicken. The halogen spotlights are easily visible in all images. In (**d**) a molehill, a rabbit, a chicken and three halogen spotlights are marked.

Each recording starts at three meters followed by an increase in height of up to 25–35 m and then back again. The telescopic boom alternates the height position of the camera, while keeping the scene within the image frame. The use of a lift instead of an actual UAV results in less motion blur. The data used in this paper consist of a total of 3987 frames with the presence of animals (rabbit and chicken), together with other hot objects (halogen spotlights, molehills, wooden poles, *etc.*). Animals were able to move within in a certain area due to fixation by a 30-cm leash. In Figure 2 the same scene is captured from 5 m, 15 m and 30 m. All thermal images are rescaled to the same size as the RGB images.

### 2.2. Detection

The measured temperature is not the actual body temperature of the animal, as the measurement is also dependent on heating from the Sun, the insulative properties of the fur, or feather coat, and the distance between the animal and the camera [21]. These factors may vary in outdoor environments; hence, the segmentation and subsequent blob detection needs to adapt to this environment.

We use a threshold dynamically adjusted to each frame by using the median temperature *t̃* in the image, to exclude outliers. The threshold value is set by:

(1) $$th=\tilde{t}+c$$

where the constant *c* ensures that only objects that are significantly warmer than the background are detected.

### 2.3. Feature Extraction: Thermal Signatures

We propose a novel feature, extracted from the thermal images, that is invariant to translation, rotation, scale and, partly, posture.

**Figure 3.**
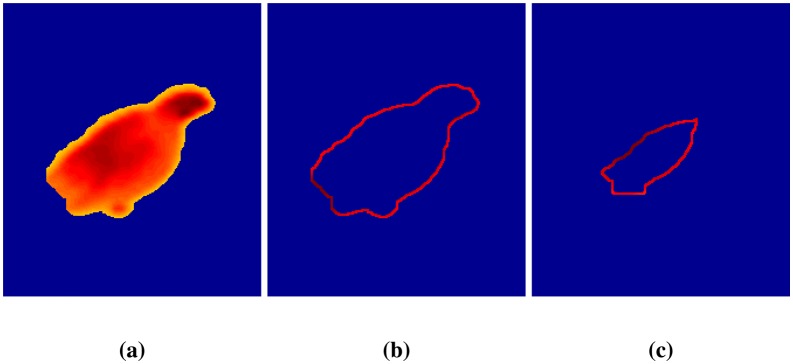
The process of extracting the thermal signature. (**a**) Thermal image of the detected object; (**b**) the first contour of the detected object; (**c**) the seventh contour of the detected object.

Based on the detected object as in Figure 3a, the perimeter contour is extracted using a four-connected neighborhood structuring element. An example of an extracted contour is shown in Figure 3b. For each iteration, the mean value of the contour is determined, and the object is shrinked by the contour. The procedure continues to iterate, until no more contours can be extracted from the object (e.g., in Figure 3b,c, the first and seventh contour are shown).

**Figure 4.**
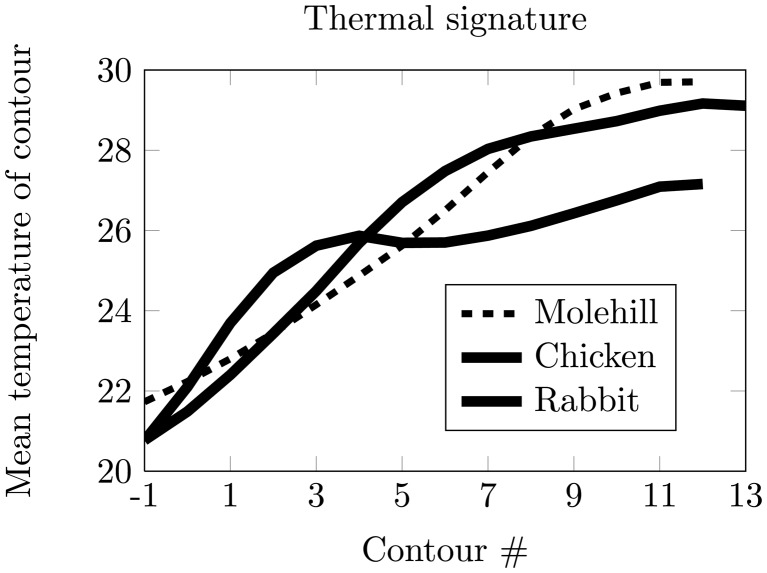
Thermal signatures extracted from shrinking thermal contours at a height of 4.9 m. Contour number *−*1 is not part of the object, but used for edge feature extraction.

The thermal signature of an object is defined as the mean thermal value of the contour in each iteration *i* and denoted as *cm*(*i*) for *i* = *−*1, …, *M*, where *M* is the maximum number of iterations possible for the given object. The first iteration is defined as *i* = *−*1, as the object is initially dilated once to get edge information just outside the object. In Figure 4, *cm*(*i*) is shown for different objects. In our dataset, a typical animal signature has a greater temperature increase close to the object boundary than a non-animal object.

#### 2.3.1. Parameterization of Thermal Signatures

The thermal signature describes certain characteristics of the objects. The signature is normalized by subtracting it with the mean temperature of the first contour. To make it invariant to the maximum number of contours, the signature can be approximated by resampling or by matching it to a high order polynomial. However, as the signature has sinusoidal characteristics, a Fourier-related transform is applicable. The discrete cosine transform (DCT) is chosen for is sinusoidal basis functions and its decorrelation properties. A fixed number of DCT coefficients will provide an approximation of the thermal signature and a set of features to be used in the classification. These feature vectors are then classified as either animal or non-animal, based on the k-nearest-neighbor (kNN) algorithm, which is briefly described in the next section.

### 2.4. Classification

The kNN algorithm is a supervised learning algorithm, which can be used for both classification and clustering [22]. When used for classification, the algorithm is based on labeled training data. We extract 140 animal-feature vectors and 359 non-animal feature vectors as training data for the kNN classifier. More non-animal data are used, as the non-animal class contains more objects with different thermal characteristics. Thus, more training data is required to model this. Based on empirical experiments, the *k* parameter was set to 11, thereby including the nearest 11 training points during classification, which is based on majority voting.

### 2.5. Classification Using Temporal Information

**Figure 5.**
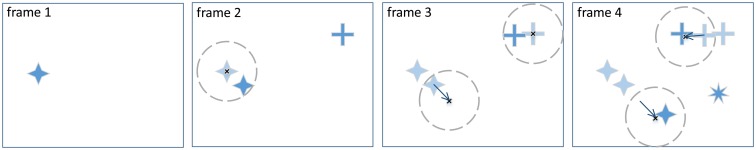
Tracking procedure.

A classification based on only a single frame using, e.g., a kNN classifier, discards the important temporal information provided in a recording. A lightweight tracking algorithm is used to link similarly positioned objects through consecutive images in the recordings. As the experiment has been done with a lift in a controlled setting, the tracking algorithm is not designed to compensate for movements of a potential UAV. To end or start new tracks, each track predicts a region defined as a guess region, where a new object needs to be positioned. An object is added to a track if it is within the guess region. A new track is created if a newly detected hot object is outside the guess region of any current tracks. A track is terminated when it fails to include any new objects for a defined number of frames. The guess region is described by a center point and a radius, where the center point is extended by the movement between the two previous objects included in the specific track. An example of the algorithm is provided in Figure 5, where tracks one, two and three are marked with ✦, ✚ and ✷, respectively.

- In Frame 1, a single object has been detected inside the frame. As no tracks have been registered, the newly detected point creates the first track, ✦.
- In Frame 2 two objects are detected. One point is within the guess region of the first track and is added to the first track. The second point is outside the guess region, and a new track is created, ✚.
- In Frame 3, new points are added to the second track. Notice that a new guess region is predicted by the previous movement, but as no animal has been detected within the guess region, no point is added to the track.
- In Frame 4, three objects are detected. Two points are added to the current two tracks, and the third point creates a new track, ✷.

Every time an object is being assigned to a certain track, the belief is updated to identify the tracked object as either animal or non-animal. The belief of track *m* is defined as the posterior probability and formulated as the probability of a detected element being an animal *A* given the newly observed data *D_n_* in frame *n*.

(2) $$Bel_{A,m}(n)=P(A|D_{n})=\frac{P(A)‥P(D_{n}|A)}{P(D_{n})}$$

The term *P* (*D_n_*) describes the evidence of the observed data. The evidence is a scale factor that ensures that the posterior probability sums to one and can be rewritten by using the law of total probability:

(3) $$P(D_{n})=P(A)‥P(D_{n}|A)+P(A^{c})‥P(D_{n}|A^{c})$$

where *A^c^* defines the non-animal objects. The term *P* (*A*) is the prior probability and describes the belief of an object being an animal before the data *D_n_* have been observed, also defined as the belief at *n −* 1.

(4) $$P(A)=Bel_{A,m}(n-1)$$

The probability *P* (*D_n_ |A*) is described as the likelihood and defined as the discriminant function *_gA_*(*D_n_*) of *kN N* given by the ratio of *k_A_* and *k*.

(5) $$P(D_{n}|A)=g_{A}(D_{n})=\frac{k_{A}}{k}$$

where *k_A_* is the number of *kN N* samples that are animals, e.g., if *k_A_* = 6 (majority vote), the probability is $P(D|A)=\frac{6}{11}\approx 0.55$.

Substituting Equations (3)–(5) into Equation (2) yields an updating scheme for every newly detected object.

(6) $$Bel_{A,m}(n)=\frac{Bel_{A,m}(n-1)‥g_{A}(D_{n})}{Bel_{A,m}(n-1)‥g_{A}(D_{n})+Bel_{A^{c},m}(n-1){‥}_{g_{A^{c}}}(D_{n})}$$

The belief updates every time an object is added to the track, but the track is finally identified as an animal if the belief exceeds 0.5. The prior probability of the first object in a track (n = 1) is set to *Bel_A,m_*(0) = 0.5. The belief has a high chance of getting stuck in zero or one if the classifier returns, respectively, zero and one. To avoid this, the classifier will, as a minimum, return 0.05 and maximum 0.95.

The algorithm for tracking objects and building belief is fit for detecting animals in large fields using a UAV. The scenario is as follows: The UAV detects hot objects at high altitudes, thus allowing the UAV to cover large areas in a short time. Due to limited resolution, the detected objects are both small and almost uniform in thermal signature at high altitudes. As presented in the results section, this affects detection and recognition performance.

Therefore, the UAV should approach the objects to increase thermal image quality with respect to classification. By using the tracking algorithm, the belief is constantly calculated. Based on this temporal update of the belief, the algorithm can classify a detected object as an animal or a non-animal.

**Figure 6.**
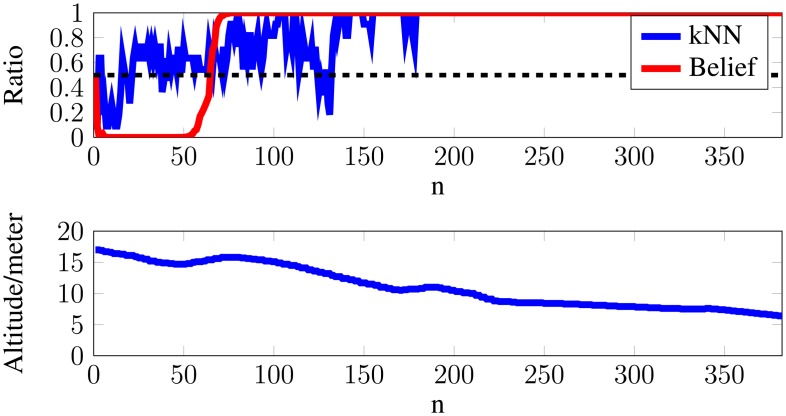
Building a belief for decreasing altitudes.

In Figure 6, the uppermost plot presents the kNN ratio from Equation (5) and the belief from Equation (6), which should be read as 1 = *animal* and 0 = *non-animal*. The bottom plot shows the altitude of the recording rig. The example shows how the belief of an object evolves as the altitude decreases. In the example, it is seen that the algorithm believes that the detected object is non-animal. However, as the belief updates, the algorithm discards this when the altitude decreases.

## 3. Results

### 3.1 Detection

Objects are detected using the threshold set by Equation (1). The parameter *c* is set to *c* = 5 ^°^*C* based on empirical experiments. The detection performance is defined as the ratio between the number of objects detected by the algorithm *l_detected_* and the actual number of animals *l* found by manual labeling.

$$D_{\text{performance}}=\frac{l_{\text{detected}}}{l}$$

**Figure 7.**
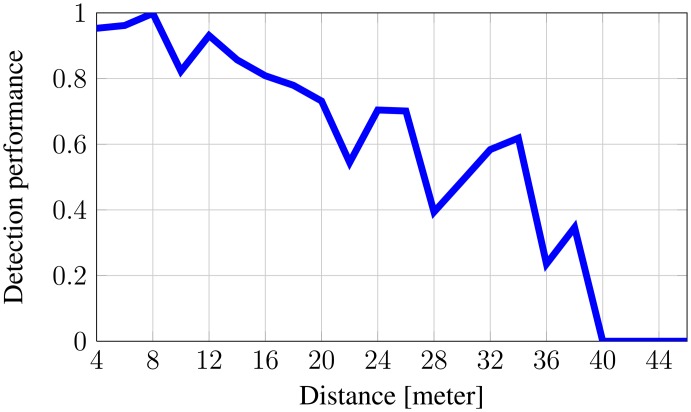
Detection performance for animals relative to altitude.

Figure 7 shows how the detection performance rapidly degrades until it reaches zero for increasing altitude.

### 3.2. Feature Extraction and Classification

**Figure 8.**
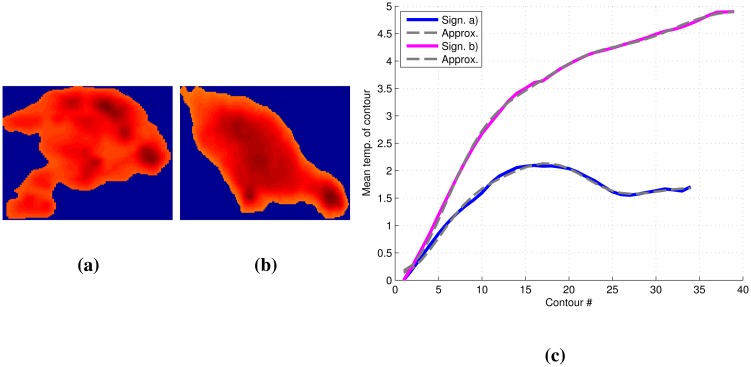
Thermal images and approximations of the thermal signature of a rabbit and chicken. (**a**) Thermal image of a rabbit; (**b**) thermal image of a chicken; (**c**) thermal signature and its seven discrete cosine transform coefficient approximation.

The thermal signature is approximated using seven DCT coefficients, as this describes 95% of the signature information for more than 95% of the provided data. Figure 8 presents an approximation of the thermal signature for two objects using seven DCT coefficients.

**Figure 9.**
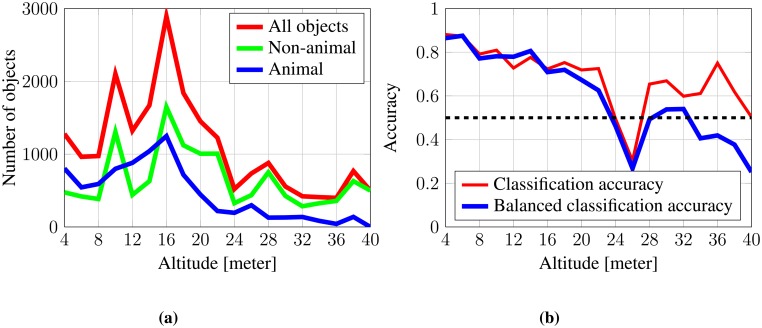
Evaluation of the classifier relative to altitude. (**a**) The number of detected objects, animals and non-animals relative to altitude; (**b**) the classifier performance using classification accuracy and balanced accuracy relative to altitude.

The classification accuracy is a common measure for classifier performance, but as presented in Figure 9a, fewer animals are detected by the segmentation algorithm for increasing altitudes. The loss of detected animals will make the data unbalanced, as it becomes dominated by non-animal samples in high altitudes.

To adjust the unbalanced data, a balanced classification accuracy is used to evaluate the classifier performance:

$$C_{\text{accuracy}.\text{balanced}}=\frac{\text{sensitivity}+\text{specificity}}{2}=\frac{TP/(TP+FN)+TN/(FP+TN)}{2}$$

where TP, FN, TN and FP are, respectively, true positive, false negative, true negative and false positive. Figure 9b shows the balanced accuracy and how performance degrades for increasing altitudes. The figure also shows that the algorithm is not able to provide satisfactory results for altitudes above 22 m, as the balanced accuracy drops below or around 0.5.

As the segmentation and classification are highly dependent on altitude, the classification is evaluated in the two different altitude ranges of 3–10 m and 10–20 m, defined as, respectively, the short- and far-range altitudes.

### 3.3 Tracking

The tracking algorithm has been setup to allow tracking of an object with three missing points and a maximum uncertainty of 190 pixels. The tracks are identified and labeled as animal or non-animal based on the updating scheme from Equation (6). After a track has been identified, all other objects in the track are changed to the similar label.

#### 3.3.1. Short Range Altitudes (3–10 m)

In the altitude range of 3–10 m, the tracker distributes 4173 out of 4381 objects (95.3%) into tracks containing more than five points, where 4104 out of 4173 objects (98.3%) are placed in a track with the majority of the same label, meaning that 1.7% objects are placed in the wrong track. The balanced classification accuracy is 84.8% before the tracks have been identified, e.g., only kNN classification is performed. Combining the classification results from each frame with the temporal information in terms of tracks, the balanced accuracy is improved by 8.7 percentage points to 93.5%. The confusion matrix before and after tracking is provided in Tables 1 and 2. Table 3 shows different performance measures with and without tracking. Sensitivity or the true positive rate (TPR) describes the classifiers ability to identify an animal object correctly. Specificity or the true negative rate (TNR) describes the classifiers ability to identify a non-animal object correctly. After tracking, the TPR and TNR are 90.8% and 96.2%, respectively, indicating that the classifier has an advantage when classifying non-animal objects.

#### 3.3.2. Far-Range Altitudes (10–20 m)

At an altitude of 10 to 20 m, the tracker distributes 8024 out of 8456 objects (94.9%) into tracks containing more than five points, where 7673 out of 8024 objects (95.6%) are placed in a track with the majority of the same label, meaning that 4.4% are placed in the wrong track.

The balanced classification accuracy is 75.2% before the tracks have been identified, while the balanced accuracy improves by 2.5 percentage points to 77.7%, when tracks are being identified. The confusion matrix before and after tracking is provided in Tables 4 and 5. Table 6 shows different performance measures with and without tracking. After tracking, the TPR and TNR are 63.4% and 90.2%, respectively, indicating that the classifier especially has difficulties classifying animal objects correctly in far-range altitudes.

The results show that information from consecutive frames in terms of determining and identifying tracks will improve performance by 8.7 and 2.5 percentage points for the short- and far-range altitudes, respectively. The system performs best in close-range altitudes with an accuracy of 93.5%, providing a lead of 15.8 percentage points compared to the far altitude range. The system maintains, though, a low number of FP or a high TNR of, respectively, 96.2% and 92.0% for short and far altitudes, meaning that the system preserves the ability to classify non-animals correctly in both ranges. Conversely, the TPR drops from 90.8% to 63.5%, meaning that the classifier especially has difficulties in recognizing animal objects correctly in far-range altitudes.

## 4. Discussion

The presented feature extraction and classification scheme shows good detection and classification performance for recording heights under 10 m with a balanced classification accuracy of 84.8%. In the altitude range of 10–20 m, the performance drops, having a balanced classification accuracy of 75.2%. The procedure becomes unfit for altitudes above 20–22 m, as detection performance decreases, but the altitude limit is ultimately set by a bad recognition, as the balanced classification accuracy drops below or around 0.5. Multiple arguments demonstrate that the application degrades for increasing altitudes, ultimately making it unfit for detecting and classifying small animals in altitudes above 20 m. The decreased detection relative to altitude is explained by the following reasons:

(1) The thermal radiation received by the sensor decreases as the distance to animal increases.
(2) The size of an animal is decreased for increasing altitudes, allowing the animal to be dominated by its colder surroundings.
(3) For a given image resolution and FOV, the spatial resolution or ground sample distance will, above a certain altitude, exceed the size of the animal, making it undetectable for the thermal imaging sensor.

**Figure 10.**
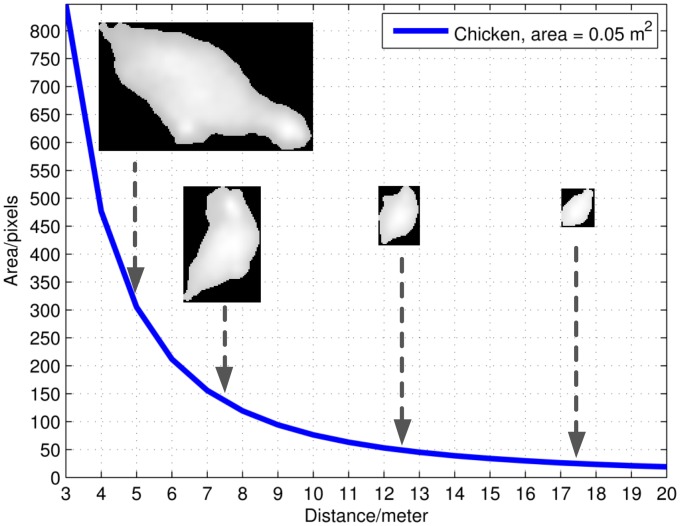
The pixel area relative to the distance for a ground area of 0.05 m^2^. Thermal images of a rescaled chicken from different altitudes.

The drop in classifier performance for increasing altitudes is explained by the increasing ground sample distance, causing the object to be presented in lower resolution or by less information, e.g., the area of a chicken (around 0.05 m^2^) will, from an altitude of 5 m, theoretically be presented by 305 pixels, while the same chicken is presented by only 19 pixels from an altitude of 20 m. Figure 10 shows how the pixel area of a chicken theoretically decreases relative to altitude and how a chicken, in practice, ends up losing characteristics.

Performance can, though, be improved in high altitudes for both detection and classification by using a higher resolution camera, a more narrow FOV or optical zoom. The decrease in performance for increasing altitudes fits well with observations from [5], where the authors were able to manually detect row deer fawns at 30 m, but had problems at 50 m with a thermal camera with a resolution of 640 × 512 pixels. The animals used in this paper are smaller than roe deer fawns, which results in fewer thermal pixels, compared to the roe deer fawns.

Tracking objects in subsequent images enables us to exploit the temporal information in the recording and improve performance. The proposed tracking algorithm improves the balanced accuracy by 8.7 percentage points to 93.5% in short-range altitudes and by 2.5 percentage points to 77.7% in far-range altitudes. A lightweight tracking algorithm has been applied to simply prove how performance can be improved by exploiting the temporal data. Tracking should, in a real application, handle larger movements in the horizontal plane and could be combined with a gimbal to stabilize the camera, independent of yaw, roll and pitch.

The manually-extracted training data is based on two types of animals: rabbits and chickens. However, other animals are of interest within the scope of wildlife-friendly agriculture. More experiments, including different weather conditions, vegetation, animals and more non-animal candidates to extend the variation of our somewhat limited dataset, should be conducted. These experiments could help improve the existing algorithm or increase our knowledge of using thermal cameras for automatic detection and recognition of wildlife. Furthermore, the applicability of the used methods should be evaluated using footage taken from an actual UAV in motion to include the effects of wind, UAV movements, moving animals and to more easily extend the variety of the dataset.

The set used for the testing and training of the classifier has no overlapping data. However, as the training data have been selected from, e.g., every 50th frame in a recording, the data used for testing and training are correlated to some extent.

This paper focuses on thermal imaging and the proposed feature extraction method. However, sensor fusion, using the RGB camera, could potentially increase classification performance. Therefore, sensor fusion methods should be investigated to accomplish this.

## 5. Conclusion

We have introduced a method for the automatic detection and recognition of wildlife using thermal cameras for UAV technology. Based on a dynamic threshold, hot objects are detected and subsequent feature extraction is performed. The novel feature extraction method, presented in this paper, consist of an extraction of thermal signatures for each detected object and a parameterization of this based on DCT.

Methods for classification using measurements from both single and multiple frames is presented. Combining measurements from multiple frames achieves the best performance, with a balanced classification accuracy of 93.5% in the altitude range of 3–10 m and 77.7% in the altitude range of 10–20 m, thus demonstrating a clear relationship between the performance of detection and classification relative to altitude. The simulated and limited dataset is favorable in terms of performance for the given algorithms. The actual applicability of the system should therefore be determined using footage from an actual UAV. The proposed detection and classification scheme is based on top-view images of wildlife, as seen by a UAV. The use of UAV-technology for automatic detection and recognition of wildlife is currently part of ongoing research towards wildlife-friendly agriculture.

## Figures and Tables

**Table 1.** Confusion matrix before track identification in the close-range altitudes (3–10 m).

		Observation	
Prediction		Animal	Non-animal
	Animal	2056	332
	Non-animal	330	1663

**Table 2.** Confusion matrix after track identification in close-range altitudes (3–10 m).

		Observation	
Prediction		Animal	Non-animal
	Animal	2167	76
	Non-animal	219	1919

**Table 3.** Performance measure in close-range altitudes (3–10 m).

	Per for mance measur e	No tracking	Tracking
Range 3-10m	Classificat ion accuracy	0.849	0.933
	Balanced classificat ion accuracy	0.848	0.935
	Sensit ivit y, T rue posit ive rat e	0.862	0.908
	Specificit y, T rue negat ive rat e	0.834	0.962

**Table 4.** Confusion matrix before track identification in far-range altitudes 10–20 m.

		Observation	
Prediction		Animal	Non-animal
	Animal	2735	515
	Non-animal	1606	3600

**Table 5.** Confusion matrix after track identification in far-range altitudes 10–20 m.

		Observation	
Prediction		Animal	Non-animal
	Animal	2753	331
	Non-animal	1588	3784

**Table 6.** Performance measure in far-range altitudes 10–20 m.

	Performance measure	No tracking	Tracking
Range 10-20m	Classification accuracy	0.749	0.773
	Balanced classification accuracy	0.752	0.777
	Sensitivity, True positiverate	0.630	0.634
	Specificity, True negative rate	0.875	0.920
